# New Strategies for Studies With Families in Borderline Personality Disorder: Challenges and Potentialities of the Use of Genogram

**DOI:** 10.1111/jmft.70065

**Published:** 2025-08-18

**Authors:** Ana Carolina Soares Marinho, Camila Chagas, José Carlos Galduróz, Fernanda celeste de oliveira Martins Sassi, Erlei Sassi Junior, Francisco Lotufo Neto, Claudia Berlim de Mello

**Affiliations:** ^1^ Department of Psychobiology Universidade Federal de São Paulo São Paulo São Paulo Brazil; ^2^ Institute of Psychiatry Universidade de São Paulo São Paulo São Paulo Brazil

**Keywords:** borderline personality disorder, emotional relationships, family relations, genogram, methods

## Abstract

Borderline personality disorder (BPD) is a complex condition characterized by emotional instability, interpersonal difficulties, and impulsivity. Although previous studies have explored the influence of family environment on BPD manifestation, methodological gaps remain in investigating these relationships, particularly in capturing multiple perspectives within the same family. This study proposes a methodological approach to compare dyads of patients and a family member using a genogram. The research involved 12 participants (6 patients and 6 family members), analyzing their perceptions of emotional family relationships (such as risk/protective factors) and symptom severity. The results of mixed methods indicated discrepancies between patients and family members regarding two categories of emotional relationships and risk behavior: “Distant/poor” and “Physical Abuse.” This study discusses other potential uses of the genogram for capturing nuances in family relationships and new possibilities for more effective therapeutic interventions.

## 1 Introduction

Borderline personality disorder (BPD) is characterized by a persistent pattern of instability in interpersonal relationships, self‐image, and emotional regulation, alongside pronounced impulsivity (APA 2022). According to the Diagnostic and Statistical Manual of Mental Disorders (DSM‐5 TR), the diagnostic criteria for BPD involves: (1) frantic efforts to avoid real or imagined abandonment; (2) a pattern of unstable and intense interpersonal relationships marked by alternating between extremes of idealization and devaluation; (3) identity disturbance involving a markedly and persistently unstable self‐image or sense of self; (4) impulsivity in at least two areas potentially self‐damaging; (5) recurrent suicidal behavior, gestures, or threats, or self‐mutilation; (6) affective instability due to marked mood reactivity; (7) chronic feelings of emptiness; (8) inappropriate, intense anger or difficulty controlling anger; and (9) transient, stress‐related paranoid ideation or severe dissociative symptoms (APA 2022). Prevalence of BPD in different countries including United States, United Kingdom, and Netherlands ranges between 1.1% and 3.2% (Ellison et al. 2018). As a complex multidimensional disorder encompassing affective, behavior, and cognitive phenomena, with individuals often struggling with chronic emptiness and loneliness, seeking excitement to cope, BPD patients may exhibit a wide range of symptoms often associated with comorbid disorders (APA 2022; Fatimah et al. 2023; Paris 2005; Zanarini et al. 1998). Consequently, achieving an accurate diagnosis can be challenging, as it may be mistaken for other clinical conditions such as bipolar affective disorder or posttraumatic stress disorder (Gunderson 2009).

The risks associated with BPD traits and the disorder itself result from the interplay between genetic, epigenetic, psychological, social, and life experience factors (Bozzatello et al. 2021; Perez‐Rodriguez et al. 2018). Childhood traumas, such as neglect, physical or sexual abuse, significantly increase the likelihood of developing the disorder (Porter et al. 2020; Zanarini et al. 2002). It is estimated that between 30% and 90% of individuals with BPD report histories of abuse or neglect, with rates markedly higher than those observed in other personality disorders (Bozzatello et al. 2021). According to the review by Bozzatello et al. (2021), these adverse experiences, especially when perpetrated by attachment figures, may lead children to internalize blame for the maltreatment they suffer within the family. Among these, sexual abuse has been identified as the only form of maltreatment with an independent association with early BPD onset in adolescent females (Infurna et al. 2016), and it is an event that is commonly reported as having occurred within intrafamilial contexts (Australian Institute of Health and Welfare 2025; CSA 2025). Apart from abuse, Crawford et al. (2009) observed, based on empirical data, that separations lasting a month or longer between mother and child—due to hospitalization, illness, extended visits to relatives, or personal, educational, or professional demands of the mother—before the age of five are associated with borderline symptoms. Additionally, Fatimah et al. (2020) found that adult antisocial behavior, substance use disorders, and paternal borderline traits increase the risk of BPD traits only in biological children. While these findings point to genetic transmission, the study also discussed environmental transmission pathways, such as maternal BPD and maladaptive parenting (e.g., conflicts, lack of involvement, and disrespect). Such patterns have been observed in both biological and adopted children (Fatimah et al. 2020; Zalewski et al. 2014), suggesting that both genetic and environmental factors may contribute to the development of BPD traits, depending on the nature of parental psychopathology.

In addition to identifying problematic patterns in family functioning, Gunderson and Lyoo (1997) emphasized the importance of how these patterns are perceived by different family members. For instance, their study revealed that it is typically relatives of individuals with BPD who report communication and temperament/hostility issues. Moreover, there is also evidence that patients show more negative judgments about their families than their parents do (Gunderson and Lyoo 1997). Notably, compared to normative (families without a psychiatrically disturbed member) and even distressed family samples (families with crises or with a psychiatrically ill or substance‐abusing member), BPD patients perceived their families as significantly less cohesive, less expressive, and more conflicted. Moreover, whenever differences emerged among patients, parents, and the distressed group, the parents tended to view the family more positively, while the patients' perceptions were more negative than those of the distressed group. These findings suggest that perceptual discrepancies within BPD families may go beyond general family variation, potentially reflecting a specific and clinically meaningful dimension of individual variability in this population. Such findings indicate that the studies with BPD families should not focus solely on the patterns of family functioning associated with risk behaviors, but also on how these patterns are interpreted by different members. Therefore, comparing the perception of the emotional relationships of patient‐relative dyads can contribute to a better understanding of the relational functioning of BPD patients.

Research focusing on the role of family links on behavioral BPD symptoms involving families have employed various methodologies with an emphasis on self‐report scales (Bozzatello et al. 2021). For instance, Roca et al. (2024) assessed by means of three questionnaires of marital (the relationship between the parents) and parental functioning (the relationship between parents and their children) in families with offspring diagnosed with BPD. Their findings revealed notable discrepancies between parents' and offspring's perceptions of care and overprotection, with BPD patients tending to view their family environment more negatively, with the authors also highlighting difficulties in family communication. Additionally, they found a link between better marital relations and greater severity of BPD symptoms. Considering that the authors adopt a relational framework‐ emphasizing how dysfunctional tendencies in marital and parental relationships strongly influence the development of personality and mental health in children—they argue that adopting a relational approach may support more effective treatment planning by addressing the relevance of family interactions rather than only an individual perspective. Although their main focus is on relational patterns, the perceptual discrepancies reported between parents and offspring also point to the potential clinical relevance of how such interactions are subjectively experienced by different family members. On the other hand, observational studies have also been conducted, such as the study developed by Kluczniok et al. (2018), which included observing a play session between a mother with a history of BPD and her child. This study revealed a link between maternal BPD and increased hostility in a mother‐child interaction, with maternal hostility mediating the association between maternal BPD and the number of psychiatric diagnoses in the child. From a clinical perspective, these findings underscore the potential of preventive, disorder‐specific interventions, for example, those focusing on reducing maternal hostile behaviors in the context of BPD.

In this way, studies about the BPD psychopathology in the context of family environment can led to the development of interventions that consider the relatives as an important component. For instance, the Helping Young People Early (HYPE) intervention, developed by researchers in Australia and targeting young people aged 15–24 with BPD, includes psychoeducation for family members about the disorder. It also aims to promote the well‐being of both family members and caregivers (Chanen et al. 2009, 2022). Besides the improvement in psychosocial functioning, this intervention has also shown to be effective in retaining young patients with BPD in care (Chanen et al. 2022). Another example is the BRIDGE (Brief, Intensive Assessment and Integrated Formulation) intervention, developed in the UK also for young people with BPD, which incorporates collaboration with a multi‐agency team tailored to the patient, potentially involving family members and counselors (Gajwani et al. 2024). However, like HYPE, family involvement is not the primary focus of the interventions. The family's role is usually to be involved in the development of a shared formulation, that is a collaborative process focusing on the definition of main problems and therapeutic goals with the young person, when clinically appropriate (Gajwani et al. 2024). It is important to note that BRIDGE is still under investigation, and outcome data regarding its effectiveness have not yet been published. Thus, although intervention studies with families of patients with BPD have shown, for example, reductions in feelings of pain and guilt, overload, and depressive‐anxious symptoms, as well as improvements in relationship skills and family climate (Guillén et al. 2021), there remains a gap regarding how therapeutic approaches might address divergences in perception within families.

Research in intimate and family relationships highlights the impact of perception over objective reality in shaping relational outcomes. It has been shown that perceived (rather than actual) similarity between couples predicts greater attraction across relationships and this could happen due to cognitive biases and self‐esteem maintaining processes (e.g., “this person is similar to me‐ or should be”) (Montoya et al. 2008). In romantic partnerships, idealizing and being idealized are associated with reduced conflict and higher satisfaction (Murray et al. 1996). Recent findings by Salla and Feixas (2023) further underscore the clinical potential of working with interpersonal perception in close relationships. Their analysis revealed that perceived similarity with the ideal partner and accuracy in perceiving the partner's self‐image were significantly associated with relationship satisfaction. In particular, they found that a more positive view of one's partner was linked not only to greater personal satisfaction, but also to higher satisfaction reported by the partner. Building on these findings, they argue about the relevance of therapeutic strategies that explicitly address metaperceptions (i.e., “What does this person think about me?”).

Together, these findings from both clinical and relational contexts underscore the relevance of working not only with the structural aspects of family functioning, but also with how individuals interpret and make sense of their relational experiences. In the case of BPD, this perspective may offer important contributions to therapeutic approaches that go beyond individual symptom management and address the relational and perceptual context in which symptoms are expressed and maintained.

The potential role of family members in the expression of BPD symptoms may also be investigated through the use of a genogram, a descriptive graphic method of analyzing family relations which has a long history in clinical settings (Varty et al. 2023). It allows for the representation of an individual's family tree, encompassing aspects ranging from biological to sociocultural dimensions (Sourdeau 2024) and has been used in various studies providing a more in‐depth description of relational aspects, allowing the observation of unsuspected family patterns (Sitnik‐Warchulska and Izydorczyk 2018; Wendt and Crepaldi 2008). The identification of the perceptions of family relationships and of discrepancies among members is also important for research in psychiatric conditions. Despite this, few studies have included genogram as a methodological strategy for investigate the role of family relationships on the symptomatology of BPD. Dominguez et al. (2025) reported its use in a case study of an individual who suffered sexual abuse but with the only purpose to describe the family structure. Additionally, study focusing on a clinical trial to investigate the effectiveness of an intervention program also included the genogram but as a component of the sociodemographic characterization of the sample (Guillén et al. 2024). Thus, the development and use of methodological strategies that focus on family relations‐ as well as potential perceptual biases‐ may be useful for clinical and scientific contexts regarding BPD approach.

In sum, the family environment plays a pivotal role in the development and expression of BPD symptoms, not only as a source of early adverse experiences linked to risk factors such as abuse, neglect, or separation, but also as a context in which ongoing relational dynamics‐ such as communication difficulties and differing perceptions between patients and relatives‐ may influence how symptoms are experienced and maintained. And although studies with families of BPD patients have shown important results, there is still a need for methodological improvements and studies that consider the potential focus on reduction in intra‐family conflicts (Guillén et al. 2021). The main aim of this study was to investigate by means of the genogram how dyads of BPD patients and a family member (preferably a sibling or parent) identify family emotional relationships and risk behaviors (ERRB). Such data correspond to individually reported relationships and events, which may be associated with potential risks or protective factors related to the development and severity of the disorder. Additionally it was investigated the extent to which such patterns were associated to the severity of the borderline symptoms, as assessed by a questionnaire. In sum, the study introduces new mixed‐methods methodologies, highlighting the use of the genogram in research involving patients diagnosed with BPD and their families. We hypothesize that significant perceptual discrepancies will emerge between patients with BPD and their relatives regarding ERRBs. Furthermore, we propose that the genogram holds potential as a valuable clinical and research tool to explore and visualize these relational dynamics.

## 2 Method

### 2.1 Participants

The sample was composed of six females diagnosed with BPD and six family members (three males and three females) indicated by them, all ranging from 18 to 56 years‐old. The relatives included four siblings, one father, and one mother, as indicated by the patients. The diagnosis was confirmed by at least two specialist psychiatrists and testing based on the Diagnostic and Statistical Manual of Mental Disorders (DSM‐IV) and the Borderline Symptom List (BSL‐23). The participants were recruited from Hospital das Clínicas of the Faculty of Medicine of the Universidade de São Paulo, specifically at the Integrated Outpatient Clinic. They were initially informed by the clinical staff about the research. In sequence they received a formal invitation and additional information about the study via a text message sent by the principal investigator. It was clearly stated that participation was entirely voluntary and would not affect their ongoing or future clinical care in any way. The clinical team only provided initial information about the study and had no involvement in decisions about participation. The clinical participants had been in treatment for at least a year, monitored by a physician, and participating in an operative group intervention with family members aimed at improving alexithymia and narrative skills as preparation for brief psychotherapy.

The inclusion criteria for the clinical group (BPD patients) were to have a diagnosis confirmed by the hospital team (that included psychiatrists and psychologists), to be literate, and over 18 years old. For the family group (the indicated relatives), the inclusion criteria required participants to be over 18 years old and literate. The exclusion criteria were participants who had uncorrected visual impairments, neurological conditions, or a diagnosis of intellectual disability. Additionally, participants in the family group could not have a diagnosis of BPD.

The sociodemographic and clinical profile is shown in Table 1. Regarding educational level, we observed significant differences between the groups. In the BPD group, most participants (42%) had completed high school, while only 8% had completed higher education. No participants in this group had completed postgraduate studies or elementary education only. In contrast, the family group shows a more diverse distribution: 17% had completed high school, 17% had completed higher education, 8% had completed postgraduate studies, and 8% had completed elementary education.

**Table 1:** Sociodemographic and clinical profile of the sample.

Variable	BPD (6)	Family (6)
Mean age	23.83	34.17
Median age	23.00	29.50
Age SD	2.71	16.38
Minimum/max age	20.00/27.00	18.00/56.00
Education level		
High School Complete	5 (42%)	2 (17%)
College Degree	1 (8%)	2 (17%)
Postgraduate Degree	0 (0%)	1 (8%)
Elementary School Complete	0 (0%)	1 (8%)
Gender		
Female	6 (50%)	3 (25%)
Male	0 (0%)	3 (25%)
BSL (borderline symptom list)		
Mean	1.500	0.616
Median	1.543	0.435
SD	0.545	0.483
Minimum/maximum	0.826/2.217	0.217/1.391
Frequencies of diagnosis and BSL severity		
None or Low	0 (0%)	3 (25.0%)
Mild	0 (0%)	1 (8.3%)
Moderate	4 (33.3%)	2 (16.7%)
High	2 (16.7%)	0 (0%)

*Note:* Family: member of the family (sibling or parent).

Abbreviations: BPD, borderline participant; SD, standard deviation.

In terms of clinical profile, the BPD group demonstrated greater severity of borderline symptoms, with the majority of participants classified as “Moderate” (33%) and “High” (17%). In contrast, the family group showed a predominance of cases with mild or absent symptoms, with 25% classified as “None or Low” and 8% as “Mild.” This difference suggests that the BPD group had a more severe symptomatology regarding borderline features measured by the BSL‐23, while the family group presented a more balanced distribution, with less impact from borderline symptoms.

### 2.2 Data Collection and Setting

The participants took part in a semi‐structured interview conducted by the first author, a clinical psychologist with training in Dialectical Behavior Therapy and clinical experience in psychotherapy with patients diagnosed with BPD. The aim was to construct a family genogram (Table 2). The genogram is a graphic strategy for the recording and interpretation of family data (genetic, cultural, social, etc.) with specific codes for members and relationships variables (see Figure 2 in Section 3 for details). The questions addressed topics such as the presence of risk behaviors in the family, perceptions of social support, the quality of family relationships, and sociodemographic characteristics. Table 1 shows a synthesis of the main contents of interest in the questions.

**Table 2:** Main categories addressed, and correspondent aspects analyzed in the semi‐structured interviews.

Category	Main aspects
General information	Identification of three family generations (parents, siblings, grandparents).Age, education, occupation, number of children.
Family history	Traumas and violence: events such as deaths, abuse, conflicts.
Social support	Level of closeness with family members.
	Breakups and perceived assistance.
Risk behaviors	Substance use, self‐harm, binge eating.
Perceptions and relationships	Family relationships: closeness, understanding, and help.
	Significant childhood events.

Data collection was conducted remotely using the Google Meet platform, with the screen mirroring feature of a tablet employed for the construction of the genogram. Interviews for the genogram construction were carried out separately, with one interview conducted with the patient and another with a relative. This helped to minimize biases and prevent one participant's discourse from being influenced by the other's statements. The remote format for data collection was chosen to be in accordance to the outpatient clinical procedures where patients were recruited.

All interviews were recorded and subsequently transcribed, allowing the researcher to revisit the categories during data analysis, thus promoting greater precision in interpreting the narratives. During the process, the researcher drew the genogram directly on the tablet screen, enabling the participant to follow the construction in real time and actively contribute to the organization and validation of the family schema (a ruled screen was used to facilitate the drawing).

**Figure 1:**
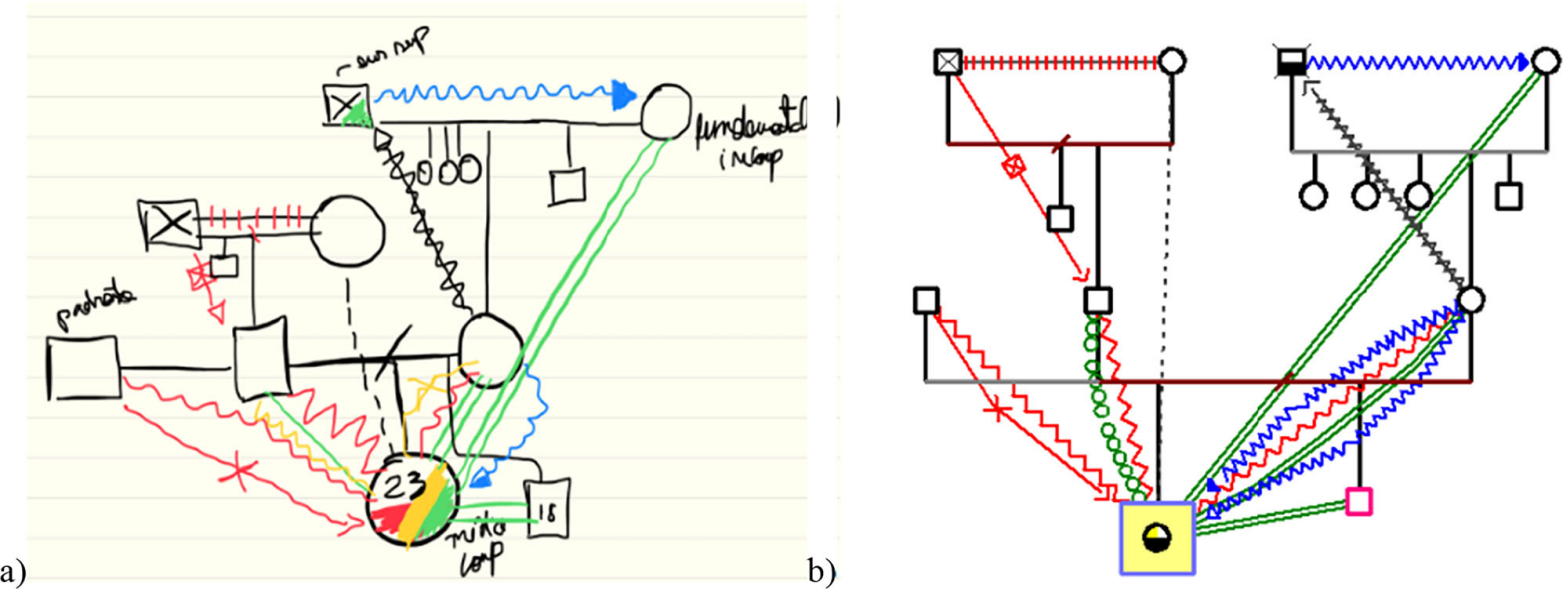
Example of a genogram constructed on the tablet (a) and transferred to the software (b).

After the initial construction on the tablet, the genogram was transferred to the GenoPro (2020) software (Figure 1), which assisted in storing and graphically representing detailed family data.

As mentioned in the introduction, the set of the representations of ERRB inferred in the analysis of participants' discourse in the genogram constructions was named in the present study as ERRB.

To classify and categorize these family relationships as defined by participants, we used the categories provided in the legend generated by the genogram software, as well as the frequency of relationships, also derived from the genogram. Each ERRB score corresponds to the frequency of each of these categorized relationships‐ that is, each event identified by each participant. In total, 28 distinct ERRB categories were identified. However, it was noted that not all relationships mentioned in the interviews, and observed in the genogram, were included in the legend provided. Therefore, the missing relationships were manually identified and classified by the researcher, ensuring that all relevant relationships were adequately considered in the analysis.

Before the interviews, participants were requested to complete the Borderline Symptom List (BSL‐23), a validated instrument designed to assess the severity of symptoms associated with BPD (Bohus et al. 2009; Catelan and Nardi 2021). Thus, based on this scale, we obtained data regarding the severity of the symptoms in the sample. We assessed these symptoms in the relative because we were interested in investigating to what extent borderline symptoms could influence the perception of ERRB characteristics. It is important to mention that the BSL‐23 assesses BPD symptoms rather than providing a clinical diagnosis. Therefore, if family members report BPD symptoms according to the scale, this does not necessarily indicate a formal diagnosis of the disorder.

### 2.3 Quantitative Data Analyses

The data were initially tabulated in Excel and later transferred to the Jamovi software (The Jamovi Project 2022) for statistical analysis. The frequency of ERRB within each family was weighted based on the number of family members present across three generations in the genogram (siblings, parents, uncles/aunts, and grandparents). In cases where there was a discrepancy between the number of family members reported by the patient and those reported by the family member, the number provided by the patient was considered, given that the study's focus was on the patient's perspective. This weighting was conducted to ensure that the quantitative analysis fairly reflected the distribution of ERRB, considering variations in the number of participants in each genogram.

To test differences in ERRB between the groups (patients with BPD and family members) and the severity of BPD symptoms, the assumptions of normality and homogeneity of variances were first examined. The Shapiro–Wilk test indicated that several variables violated the assumption of normality (e.g., Abuse, *W* = 0.520, *p* < 0.001; Distrust, *W* = 0.561, *p* < 0.001; Risky Eating Behaviors, *W* = 0.828, *p* = 0.0199). Levene's test also revealed violations of the assumption of homogeneity of variances for certain variables (e.g., Hostile, *F*(1, 10) = 10.44, *p* = 0.009; Distant/poor, *F*(1, 10) = 5.62, *p* = 0.039). Given these violations (*p* < 0.05), the nonparametric Kruskal–Wallis test was used for group comparisons.

A Kruskal‐Wallis nonparametric analysis of variance was conducted, considering the variable “diagnosis,” a categorical variable representing group membership (clinical group– individuals with BPD–or family members), and ERRB scores as the continuous dependent variable. Additionally, the same test was applied to analyze the relationship between symptom severity, a categorical variable classified into four levels (none/low, mild, moderate, and high), and ERRB.

### 2.4 Qualitative Data Analyses

For the qualitative analysis the genograms of the patient and corresponding family member were first examined individually and then compiled and integrated into a single document to allow for a comparative interpretation of relational patterns. The focus was placed on identifying if the significant ERRB highlighted in the quantitative analyses were present, and between which specific family members these relations occurred.

This approach was guided by the multiple case study methodology as described by Yin (2003), which considers each case—in our study, each patient‐relative dyad—as a “whole study” in itself. In multiple case studies, the goal is not to generalize statistically from a sample to a population, but to explore whether patterns replicate across different, purposefully selected cases. This is known as the replication logic, where each case either confirms a pattern (literal replication) or shows a contrasting result for theoretically anticipated reasons (theoretical replication) (Yin 2003). This logic is analogous to running a series of experiments, in which each case serves to confirm, disconfirm, or refine the emerging theoretical propositions.

In our study, this design allowed us to examine whether ERRB identified in the quantitative analysis were also represented in the qualitative genogram data, and how these patterns varied across families. This methodology also integrates qualitative and quantitative data, a combination highly powerful.

Following Eisenhardt (1989) recommendations for building theories from case studies, we employed both within‐case and cross‐case analyses. Within‐case analysis involved detailed exploration of each dyad's dynamics, while cross‐case comparison sought to identify patterns of similarity and difference across dyads. This tactic forces the researcher to systematically compare and contrast cases. Such comparisons are central to theory‐building because they support the development of more robust, generalizable, and empirically grounded insights (Eisenhardt 1989). Overall, this multiple‐case approach enabled us to explore the interplay between relational perceptions across families in BPD context.

## 3 Results

### 3.1 Quantitative Results

The non‐parametric analysis of variance revealed a significant difference between the groups for the emotional relationship Distant/poor, *χ*² (*df* = 1) = 5.429, *p* = 0.0198, and Physical Abuse, *χ*² (*df* = 1) = 5.133, *p* = 0.0235 (Table 3).

**Table 3:** Differences in characteristics of familial ERRB perceived by the patient and the family member.

	*χ*²	*gl*	*p*‐value	*ε*²
Distant/poor	5.429	1	**0.0198**	0.494
Friendship/close	0.234	1	0.6285	0.021
Hostile	0.000	1	1.0000	0.000
Physical abuse	5.133	1	**0.0235**	0.467
Abuse	0.015	1	0.9020	0.001
Emotional abuse	0.027	1	0.8700	0.002
Distrust	1.000	1	0.3173	0.091
Social support	0.006	1	0.9358	0.001
Risky eating behavior	2.314	1	0.1282	0.210
Risky sexual behavior	0.263	1	0.6081	0.024
Risky drug and alcohol use	0.646	1	0.4217	0.059
Suspicion of alcohol and drug use	1.000	1	0.3173	0.091
Controlling	0.739	1	0.3900	0.067
Negatively focused on	2.182	1	0.1396	0.198
Manipulation	0.707	1	0.4005	0.064
Cut/distant relationships	1.098	1	0.2947	0.100
Restored cut relationships	0.178	1	0.6733	0.016
Disagreement/conflict	0.072	1	0.7884	0.007
Harmony	0.532	1	0.4657	0.048
Violence	0.163	1	0.6863	0.015
Never met/saw	0.082	1	0.7745	0.007
Neglect	0.177	1	0.6742	0.016
Incarceration	1.000	1	0.3173	0.091
Postpartum depression	1.000	1	0.3173	0.091
Fan/admirer	1.000	1	0.3173	0.091
Love	1.000	1	0.3173	0.091
Jealousy	0.544	1	0.4606	0.049
Rape/force relationship	1.000	1	0.3173	0.091

*Note:* Nonparametric analysis of variance (Kruskal–Wallis).

The comparison of means and medians revealed that, for the emotional relationship Distant/poor, participants diagnosed with BPD exhibited lower values (mean = 0.076; median = 0.072) compared to family members (mean = 0.238; median = 0.221), suggesting that individuals with BPD perceived this relationship less frequently than family members. Conversely, for the emotional relationship Physical Abuse, individuals with BPD presented higher values (mean = 0.251; median = 0.191) compared to family members (mean = 0.061; median = 0.026), indicating that BPD patients reported a higher perception of this experience compared to family members (Table 4).

**Table 4:** Patient perception of situations in relation to their respective family members, regarding distant/poor relationships, and physical abuse.

	Group	Mean	Median
Distant/poor	BPD	0.076	0.072
	Family	0.238	0.221
Physical abuse	BPD	0.251	0.191
	Family	0.061	0.026

*Note:* Family: member of the family (sibling or parent).

Abbreviation: BPD, borderline participant.

Additionally, a nonparametric analysis of variance (Kruskal–Wallis) was conducted to investigate the relationship between the severity of BPD symptoms and ERRB. The results indicated that, overall, no statistically significant differences were found for most of the evaluated ERRB (*p* > 0.05), except for the variable Suspicion of Alcohol and Drug Use, which showed a significant difference between the groups, *χ*² (*df* = 3) = 11.00, *p* = 0.0117 (Table 5). It is important to note that this significant result is based on data from a single participant, a sister (family group) in the“mild” severity subgroup.

**Table 5:** Statistical analysis of the relationship between the severity of BPD symptoms and ERRB.

	χ²	gl	*p*	ε²
Distant/poor	4.574	3	0.2058	0.416
Friendship/close	0.650	3	0.8849	0.059
Hostile	7.294	3	0.0631	0.663
Physical abuse	4.194	3	0.2413	0.381
Abuse	2.182	3	0.5355	0.198
Emotional abuse	1.994	3	0.5737	0.181
Distrust	1.000	3	0.8013	0.091
Social support	3.343	3	0.3417	0.304
Risky eating behavior	3.182	3	0.3644	0.289
Risky sexual behavior	3.871	3	0.2757	0.352
Risky drug and alcohol use	3.986	3	0.2630	0.362
**Suspicion of alcohol and drug use**	11.000	3	**0.0117**	1.000
Controlling	5.254	3	0.1541	0.478
Negatively focused on	2.576	3	0.4618	0.234
Manipulation	4.440	3	0.2177	0.404
Cut/distant relationships	2.850	3	0.4154	0.259
Restored cut relationships	5.044	3	0.1686	0.459
Disagreement/conflict	2.430	3	0.4881	0.221
Harmony	6.742	3	0.0806	0.613
Violence	0.675	3	0.8790	0.061
Never met/saw	4.556	3	0.2074	0.414
Neglect	1.811	3	0.6125	0.165
Incarceration	5.000	3	0.1718	0.455
Postpartum depression	1.000	3	0.8013	0.091
Fan/admirer	1.000	3	0.8013	0.091
Love	5.000	3	0.1718	0.455
Jealousy	1.683	3	0.6406	0.153
Rape/force relationship	3.000	3	0.3916	0.273

*Note:* Nonparametric analysis of variance (Kruskal–Wallis).

Abbreviations: BPD, borderline participant; ERRB, emotional relationships and risk behaviors.

### 3.2 Qualitative Results

Each dyad's genogram (patient and family member) was individually reviewed and then compared with the other, based on the study objectives. This approach allowed not only the quantification of ERRB but also an understanding of how they manifested within each family unit and their comparison to the corresponding family member's genogram. Below, we present a descriptive summary of the main findings, analyzed at the individual level and across dyads, highlighting both the frequency and the relational context of distant/inferior relationships and physical abuse, as reported by each participant (Table 6).

**Table 6:** Summary of dyadic perceptions regarding “Distant/poor” and “Physical Abuse” relationships reported in genogram‐based qualitative analyses.

Family	Role	No. “Distant/poor”	“Distant/poor” – Who?	No. “Physical Abuse”	“Physical Abuse” – Who?
Fam1	Patient	1	Patient – maternal grandmother	2	Mother – patient; maternal grandfather – maternal grandmother
	Sibling	5	Patient – mother; sibling – mother; patient – paternal grandmother; patient – paternal uncle; father – sibling	2	Mother – sibling; mother – patient
Fam2	Patient	1	Patient – maternal grandfather	2	Mother – sibling; father – mother
	Sibling	4	Patient – paternal grandfather; patient – second sibling; patient – third sibling; sibling – maternal grandmother	0	—
Fam3	Patient	0	—	1	Maternal grandmother – patient
	Sibling	2	Maternal grandfather – patient; paternal grandmother – patient	1	Maternal grandmother – patient
Fam4	Patient	1	Patient – sibling	3	Maternal grandparents; paternal grandparents; patient – partner
	Sibling	2	Patient – sibling; patient – father	2	Mother – sibling; patient – sibling
Fam5	Patient	2	Patient – paternal grandmother; patient – paternal uncle	5	Patient – partner; paternal grandfather – father; maternal grandmother – patient; maternal grandmother – mother; paternal grandfather – patient
	Mother	3	Paternal grandfather – father; paternal grandmother – father; paternal grandmother – patient	0	—
Fam6	Patient	0	—	4	Paternal grandfather – father; father – patient; mother – patient; patient – maternal aunt
	Father	1	Patient – sibling	0	—

## 4 Discussion

In this study, we investigated similarities and discrepancies in the perception of ERRBs according to the perspective dyads of an individual with borderline personality disorder and a family member. For this purpose we analyzed the associations among its components and two outcomes, diagnosis and the severity of symptoms.

We found differences in the perception of two categories of ERRB between the BPD group and the family group: Distant/poor and Physical Abuse. In the ERRB framework, Physical Abuse refers to a relationship in which one individual physically abuses another, including any nonaccidental injuries such as hitting, kicking, slapping, burning, or choking. The Distant/poor category, on the other hand, describes a relationship marked by limited communication, often due to perceived differences in lifestyle (Emotional Relationships—GenoPro 2025). Physical abuse was more frequently reported by the clinical participants, while the category distant/poor was more frequently reported by relatives. Additionally, our results suggest that the perception of suspicion of alcohol and drug use may be associated with the severity of BPD symptoms, since other dimensions of ERRB did not show a significant association. However, it is important to highlight that this ERRB was reported by only one participant, who belonged to the “mild” severity group—a sister who described the suspicion of alcohol and drug use regarding the patient. While this single report does not allow for statistical inference, we consider it noteworthy from a clinical and exploratory perspective, especially given the aim of this study to introduce a new approach to assessing emotional and risk‐related family dynamics in BPD. Such individual perceptions, though not generalizable, may still offer valuable input for therapeutic assessment and intervention planning. It seems, therefore, that reports of such ERRB during the genogram construction could be useful to a better understanding of the individual variability that may be associated with symptom severity and relational difficulties. They may also reflect specific biases and communication patterns within both the patient itself and the family system, reinforcing the importance of not only examining objective patterns of family functioning, but also understanding how these patterns are subjectively interpreted by each member. Exploring both similarities and differences in these perceptions may offer insights into how perceptual biases and communicative patterns shape the experience of emotional relationships within the family, revealing distortions or mismatches that could otherwise remain undetected.

Our quantitative analysis revealed that patients reported the emotional relationship of “physical abuse” more frequently compared to the family group. However, by integrating qualitative analysis, it was possible to identify which family members were mentioned in reports and to verify whether there was a consistency between the patient's and the family member's accounts. We believe that this combined methodological approach allows for a deeper understanding of BPD individual perceptions and possible divergences and alignments in the experience of ERRB within the family.

**Figure 2:**
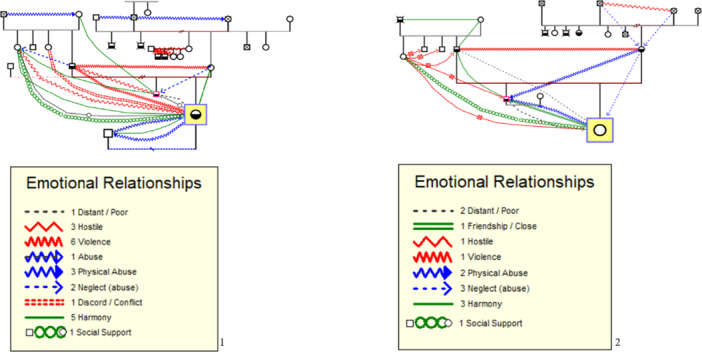
Examples of two genograms created remotely. Number 1 reflects the patient's perception, and Number 2 reflects the sibling's perception. *Patient; BSL‐23 = moderate. Sibling; BSL‐23 = moderate.*

In the example presented in Figure 2, two members of the same family‐ a patient and a family member, in this case, a sibling‐ were analyzed. Although the quantified legend generated by the genogram provided valuable information about the ERRB and their frequency, the graphical representation of the genograms enabled a more detailed analysis. Through this visualization, it was observed that the patient reported a physical abuse relationship between their paternal grandparents, whereas their sibling described the relationship of the same couple as one of harmony. Although both genograms belong to the same family, each one reflects the unique perspective of an individual (one being the patient and the other a family member), which can result in differences in the representations. For example, one line may vary because each person perceives and interprets family relationships differently.

Given the presence of perceptual discrepancies, interventions that incorporate family members, such as family therapy, may help promote emotional attunement, reduce conflict, and improve overall treatment outcomes. While family involvement is not always central in existing BPD interventions, our results suggest that therapeutic approaches addressing family communication and interpersonal perception may represent an important complement to individual treatment strategies. Identifying how patients and relatives perceive emotional closeness, conflict, or abuse can offer new insights into relational dynamics that may influence symptom expression or maintenance.

Thus, incorporating elements that explore how individuals believe they are perceived by others (metaperception) may further enhance the effectiveness of family‐based interventions. Given the potential influence of cognitive biases and self‐esteem maintaining processes on interpersonal perception (Montoya et al. 2008)—as well as the role of idealization and being idealized in regulating conflict—therapeutic strategies that help clarify mutual perceptions and foster constructive reinterpretation of relational experiences may be beneficial (Murray et al. 1996). Interventions that include circular questioning, shared formulation, or qualitative tools such as relational grids (Salla and Feixas 2023)—such as family therapy—could be adapted to facilitate this process, helping family members understand not only each other's experiences, but also how those experiences are subjectively constructed.

This perspective also reinforces the value of interventions that facilitate open communication within families in clinical settings. The Sibling (SIBS) intervention, developed in Norway, for example, aims to strengthen communication between parents and siblings of children with chronic disorders while empowering parents to provide both informational and emotional support (Haukeland et al. 2020). This intervention showed significant improvement in the quality of the dyad communication, along with reported enhancements in mental health outcomes among siblings. Given that our study highlights differing perceptions of family dynamics in BPD, an intervention that fosters emotional sharing and improves mutual understanding, in the BPD context, could help bridge these perceptual gaps.

In sum, our findings support a relational and perception‐informed approach to treatment planning in BPD. Future interventions could benefit from drawing on insights from relational and metacognitive therapy models to bridge gaps in understanding and foster more coherent and validating family environments.

Additionally, when considering the development of interventions focusing on communication among family members for BPD, it is essential to better evaluate the specific communication deficits involved in BPD psychopathology and within each family. Thus, improving the instruments and methods capable of addressing these objectives become crucial, especially as self‐report scales often fail to capture the patient's discourse in a more open‐ended manner, overlooking critical aspects of the individual's subjectivity. Therefore mixed methods seem to be more appropriate to better development of intervention strategies or specialized protocols. Being thus, the use of the genogram seems to be a methodological alternative for studying families in the context of BPD.

To our knowledge, this is the first study to use the genogram as a method for evaluating the perception of “duplicated” family trees within families, and as such, it presents strengths and challenges. The genogram allows the participant the opportunity to follow the construction of their family genogram in real‐time, which enabled them to contribute more actively to the process by making observations and comments they deemed relevant. Operating in this way, reduced the chance of the researcher misinterpreting what was being said by the interviewee. Furthermore, the use of genograms may represent a valuable addition to clinical practice, particularly as a tool to visualize relational dynamics and elicit discussion about conflicting narratives. Through the genogram, it is possible to integrate the individual's discourse obtained via a semi‐structured interview while quantifying reports regarding emotional relationships and risk behaviors. By mapping both objective events and subjective perceptions, genograms can help clinicians identify areas of miscommunication, unacknowledged conflict, or relational asymmetries that may be relevant for treatment planning. Future studies using such methodological strategy may contribute to enhance the external validity of results and integrating genogram‐based assessment into psychoeducational programs or family.

Few studies, like ours, have employed quantitative analyses in the context of genograms. One example is the study by Sitnik‐Warchulska and Izydorczyk (2018) which, like ours, explored family patterns but in a nonclinical context‐ and in an individual perspective other than dyadic. Their aim was to identify family configurations that warrant therapeutic attention due to their association with suicidal or violent behaviors in adolescent girls. Through genogram‐based analysis, the authors found that such behaviors were linked to cross‐generational family stressors‐ such as emotional difficulties, chronic illness, and triangulated relationships—and emphasized that these configurations could serve as clinically relevant indicators. Sitnik‐Warchulska and Izydorczyk (2018) underscore that recognizing these patterns is valuable in clinical practice and can be helpful in a prevention context. These findings support the relevance of genogram‐based approaches for identifying dysfunctional family dynamics, which is also a central objective of our study. While Sitnik‐Warchulska and Izydorczyk (2018) conducted their research in person, our study was carried out remotely. Consistent with this idea, samples collected through remote research may exhibit greater racial and geographical diversity than in‐person studies (Shields et al. 2021). Moreover, the remote use of the genogram also presented other significant advantages, particularly in research involving families, as it enables the inclusion of participants who, for various reasons, may not be available for in‐person assessments, whether it is the patient or the family member. This approach broadens research possibilities and facilitates data collection in an accessible and flexible format. In addition, it is possible that being at home, the participant feels more comfortable discussing sensitive topics.

To the best of our knowledge, only one published study has incorporated the remote construction of a genogram as part of its methodology, not limited to borderline personality disorder. Andrade et al. (2024) developed participants' genograms during remote interviews using screen mirroring with the GenoPro software (GenoPro 2020). However, we did not find any study, either remote or in‐person, that utilized genograms in the context of BPD.

Finally, remote research has the potential to overcome certain barriers but may also introduce new challenges. A crucial aspect that can impact the construction of the genogram is cultural influence, as different words can carry distinct meanings for everyone. For instance, the term “abuse” may be interpreted in various ways by participants, depending on their experiences and cultural contexts. Therefore, it is essential for researchers to remain attentive to ensure a proper understanding of what the interviewee intends to convey. To mitigate this challenge, it is important to involve other researchers in categorizing the topics covered (Carter et al. 2014). Reviewing transcripts with input from multiple professionals can help minimize interpretative biases and ensure a more accurate and contextualized analysis of the collected narratives.

Some limitations can be identified in this study. First, the sample was recruited in a single outpatient clinic where patients were already under treatment, and therefore they may represent a profile of patients with better clinical outcomes. Additionally only one relative for each patient was included in this study. The isolated perspective of a single relative with possible close relationships, especially as they were referred by the patient, may result in bias in family perception. Finally, the very small sample limits our conclusions. However, this limitation is consistent with the study's exploratory nature and methodological focus. For instance, although the kinship between each participant with BPD and their respective family member (e.g., mother, father, and sibling) was identified and represented in the genogram following standard conventions, these specific familial relationships were not included in the quantitative analyses. This decision was based on the small sample size and the primary objective of the study, which was to present the genogram as a methodological tool for mapping ERRB, rather than to examine differences based on the type of family relationship. Studies involving larger samples with more diverse contexts, such as different age groups and individuals without prior treatment, are required to ensure that the results are more representative. It should also be noted that remote data collection may be very challenging due to the diversity of family dynamics and routines at home that mean it may be difficult for the participant to have complete privacy. This study focuses on the subjective perceptions of each participant regarding their family relationships. Therefore, differences in accounts‐ such as a parent not recognizing behavior perceived as abusive by the other‐ are expected and meaningful. Rather than being seen as methodological bias, these discrepancies reflect interpretative divergences that are central to the understanding of relevant emotional relationships and risk behaviors.

## 5 Conclusion

In conclusion, the use of the genogram seems to be an appropriate methodological‐clinical strategy for studying families with individuals with borderline personality disorder. It allowed convergences and discrepancies in family perceptions about emotional relationships and risk behavior to be identified, specific differences in these perceptions to be analyzed, and also to identify which specific family members were highlighted in each situation. By comparing the representations of the same relationships from two different perspectives, the study offers insights into how perceptual divergences may reflect individual variability, perceptual biases, and communication patterns characteristic of BPD family dynamics. This comparison contributes to the development of more personalized interventions, especially regarding the identification of target ERRB and the most affected family members. Thus, the genogram may serve as a valuable tool not only for research but also for therapeutic assessment and intervention planning. Its remote application can facilitate sample diversity, making data collection more flexible and accessible. The use of the genogram stands out as an innovative procedure in respect of research and the clinical approach to the treatment of individuals with BPD, providing a structured means to explore family dynamics and improve future interventions.

## Ethics Statement

This study was approved by the Ethics and Research Committee of the Universidade Federal de São Paulo, under opinion number 6.957.080. All participants signed an Informed Consent Form before participation. This study was conducted in accordance with the Declaration of Helsinki.

## Data Availability

Interview data cannot be publicly shared due to confidentiality. All other data described in this manuscript are available at: https://osf.io/um4r9/?view_only=997d8e5521b34fcb8f9226280e5ed448.
